# *IAP*s Gene Expansion in the Scallop *Patinopecten yessoensis* and Their Expression Profiles After Exposure to the Toxic Dinoflagellate

**DOI:** 10.3389/fphys.2021.633301

**Published:** 2021-02-05

**Authors:** Xiaomei Zhu, Fengmei Zhang, Shanshan Lian, Yinghui Wang, Naina Hu, Xiaomei Chen, Xiaoting Dai, Xiaoli Hu, Shi Wang, Zhenmin Bao

**Affiliations:** ^1^MOE Key Laboratory of Marine Genetics and Breeding, Ocean University of China, Qingdao, China; ^2^Laboratory for Marine Biology and Biotechnology, Pilot Qingdao National Laboratory for Marine Science and Technology, Qingdao, China; ^3^Laboratory for Marine Fisheries Science and Food Production Processes, Pilot Qingdao National Laboratory for Marine Science and Technology, Qingdao, China

**Keywords:** *Patinopecten yessoensis*, IAPs, gene expansion, PST exposure, expression regulation

## Abstract

Inhibitors of apoptosis proteins (IAPs) are conserved regulators involved in cell cycle, cell migration, cell death, immunity and inflammation, should be due to the fact that they can assist with the ability to cope with different kinds of extrinsic or intrinsic stresses. Bivalve molluscs are well adapted to highly complex marine environments. As free-living filter feeders that may take toxic dinoflagellates as food, bivalves can accumulate and put up with significant levels of paralytic shellfish toxins (PSTs). PSTs absorption and accumulation could have a deleterious effect on bivalves, causing negative impact on their feeding and digestion capabilities. In the present study, we analyzed *IAP* genes (*PyIAP*s) in Yesso scallop (*Patinopecten yessoensis*), a major fishery and aquaculture species in China. Forty-seven *PyIAP*s from five sub-families were identified, and almost half of the *PyIAP* genes were localized in clusters on two chromosomes. Several sites under positive selection was revealed in the significantly expanded sub-families BIRC4 and BIRC5. After exposure to PST-producing dinoflagellates, *Alexandrium catenella*, fourteen *PyIAP*s showed significant responses in hepatopancreas and kidney, and more than eighty-five percent of them were from the expanded sub-families BIRC4 and BIRC5. The regulation pattern of *PyIAP*s was similar between the two tissues, with more than half exhibited expression suppression within three days after exposure. In contrast to hepatopancreas, more acute changes of *PyIAP*s expression could be detected in kidney, suggesting the possible involvement of these *PyIAP*s in tissue-specific PST tolerance. These findings also imply the adaptive expansion of bivalve *IAP* genes in response to algae derived biotoxins.

## Introduction

Apoptosis is a form of programmed cell death, which can be triggered by intrinsic cellular genotoxic stress, or by extrinsic signals such as the ligand binding with cell surface death receptors (Pistritto et al., 2016). In cope with different kinds of stresses, organisms could recruit multiple apoptosis regulators in order to balance between the pro- with anti-apoptotic mechanisms, inhibitors of apoptosis proteins (IAPs) for example (Grzybowska-Izydorczyk and Smolewski, 2008). IAPs, also known as baculovirus IAP repeat (BIR)-containing proteins (BIRCs), are functionally conserved in metazoans (Fulda and Vucic, 2012). *IAP* homologous genes could be widely found in insects, birds, fishes, mammals, and viruses (Luque et al., 2002), however, the *IAP* gene family vary a lot in gene numbers among species. For instance, in human, a total of eight IAP members known as BIRC1-8 were identified through functional screening and homology searching (Salvesen and Duckett, 2002; Vucic, 2008). In comparison, there are only seven, four and three *IAP* members were found in sea urchins (Robertson et al., 2006), fruit flies (Jones et al., 2000; Domingues and Ryoo, 2012), sea anemone (Sullivan et al., 2006), respectively, and more than forty *IAP* members were reported in bivalve mollusc *Crassostrea gigas* (Zhang et al., 2012), suggesting the adaptive lineage-specific gene gain and loss events.

As their functional diversity and importance are concerned, *IAP*s have received substantial research interests in a lot of animal species, related to cell fate control as well as body immunity and inflammation. For example, in human, transduction with an adenovirus encoding either *HIAP1*/*c-IAP2* or *HIAP2*/*c-IAP1* was found be able to suppress ER stress-induced apoptosis (Cheung et al., 2006). In the context of a highly inflammatory virus infection environment, *IAP*s were found could play a crucial role in the expansion of T-cells in mice (Gentle et al., 2014). Similarly, *DIAP2* was found functioned in the host immune response to gram-negative bacteria in *Drosophila* (Leulier et al., 2006). Besides, it was reported that vertebrate *IAP*s could participate in coping with toxic stresses. In human, *IAP*s directly ubiquitinate Rac1 at lysine 147 resulting in its proteasomal degradation under CNF1 toxin treatment (Oberoi-Khanuja and Rajalingam, 2012). Meanwhile, in mice, the survivin (BIRC5) was proved to serve as a protector against toxin-induced acute renal failure and *IAP*s were associated with T-2 toxin-induced mouse chondrocyte damage (Kindt et al., 2008; Wang et al., 2020).

Marine bivalves are recognized for their well adaptation to the highly complex aquatic-environment (Wang et al., 2017). These filter-feeding bivalves can usually accumulate paralytic shellfish toxins (PSTs) by filtering toxic algae or their cysts, especially during the outbreak of red tide (Yang et al., 2018). PSTs, mainly produced by toxic dinoflagellate such as *Alexandrium* (Anderson et al., 2012), are one kind of potent neurotoxins with inhibitory effects on cell sodium channels (Cusick and Sayler, 2013). PSTs accumulated in bivalves can exert negative impact on their biological function and its sanitary quality, bringing huge economic losses in the aquaculture sector (Landsberg, 2002; Contreras et al., 2011; Vilarino et al., 2013; Borcier et al., 2017). Researchers revealed that the induction of toxic stress by PST accumulation may impact on cellular apoptosis process and immune defense. It has been found that PST could enter the cytoplasm and induce apoptosis of immune cells through a caspase-dependent pathway in oyster (Abi-Khalil et al., 2017). Cellular immunological and inflammatory responses were observed after exposure to *Alexandrium catenella* in Surf clams *Mesodesma donacium* (Alvarez et al., 2019) and hemocyte aggregation were found show up after exposed to the PST-producing *Gymnodinium catenatum* in scallop *Nodipecten subnodosus* (Estrada et al., 2007). Especially, it has been found that *CgIAP1* and *CgIAP7B* were involved in the regulation of hemocyte immune cell apoptosis with toxic diet *A. catenella* (Medhioub et al., 2013), implying the possible participation of *IAP* genes in bivalves facing toxic challenge. However, to our best knowledge, systematic studies on bivalve *IAP*s in coping with PSTs stress have not been reported yet, and relevant research data may help in-depth understanding of their adaptive features to algae derived toxins.

Yesso scallops (*Patinopecten yessoensis*) are important fishery and aquaculture species in China, and they are also one type of bivalves that possess outstanding capacity to accumulate and tolerate PSTs (Cheng et al., 2016; Xun et al., 2020). According to our previous work, we found the hepatopancreas and kidney in scallops should be the top-ranked toxin-rich or toxin-tolerant organs, with hepatopancreas mostly accumulating the incoming toxins, and the kidney transforming and/or eliminating them (Tian et al., 2010; Li et al., 2017). In the present study, we provided a systematic characterization of *IAP* genes in *P. yessoensis*. In the two most toxic organs, hepatopancreas and kidney, their transcriptional responses to the exposure of PST producing algae *A. catenella* (strain ACDH) were also analyzed. Our findings will provide helpful resources for further researches to elaborate the functions of the bivalve *IAP*s and assist in better understanding of the involvement of *IAP*s in the adaptive response to PST tolerance.

## Materials and Methods

### Genome-Wide Identification and Characterization of *IAP* Genes in *P. yessoensis*

The genome and transcriptome sequences of *P. yessoensis* (Wang et al., 2017) were searched against the available *IAP* sequences from representative species, including *Homo sapiens*, *Xenopus laevis*, *Gallus gallus*, *Danio rerio*, *Mus musculus*, *Lottia gigantean*, *Octopus bimaculoides*. We downloaded the orthologous *IAP* sequences of these representative species from NCBI^1^ and Uniprot^2^ databases (Supplementary Table 1 shown the accession numbers), and used these sequences as queries to perform whole-genome and transcriptome-based blast with the e-value threshold of 1E-05. To ensure the completeness of *IAP* genes, TBLASTN with the domain sequences of all known *IAP* proteins were used as queries for additional similarity searches against genomes. Then, BIR HMM searching method was used for sequence analysis to confirm the completeness of *IAP*s, and the number of conserved BIR Pfam domain (PF00653) (Finn et al., 2016) was summarized for *P. yessoensis IAP*s in Supplementary Table 2. Next, we used open reading frame (ORF) finder program^3^ to translate candidate *PyIAP*s into amino acid (aa) sequences. The translated sequences were submitted to the SMART program^4^ to verify the presence of the BIR domain and confirm the accuracy of the sequence. The information of isoelectric point (PI) and molecular weight were predicted through Compute PI/Mw tools^5^. The genetic structure of all the identified *PyIAP*s and their protein structures were drawn with GSDS2.0^6^ and IBS1.0.3 software (Liu et al., 2015), respectively, and the genomic distribution of *PyIAP*s were also confirmed.

### Phylogenetic Analysis

The whole amino acid sequences of *IAP* genes from zebrafish (*Danio rerio*), frog (*Xenopus laevis*), original chicken (*Gallus gallus*), human (*Homo sapiens*), mouse (*Mus musculus*), lizard (*Lottia gigantean*), octopus (*Octopus bimaculoides*) were downloaded from the NCBI (Supplementary Table 1). Multi-sequence alignment for the BIR domains was performed using ClustalW (Larkin et al., 2007) as well as Genedoc software (Nicholas et al., 1997). The phylogenetic analysis was generated through MEGA6 (Tamura et al., 2013), and a neighbor-joining (NJ) tree was constructed using the p-distance method and maximum likelihood (ML) method. The gaps and missing data in positions were eliminated, and we tested the robustness of phylogeny with a bootstrap of 1000 replicates. The resulting phylogenetic tree was output in Newick format, and the online software iTOL^7^ converted the percentage value to 0-1, and scores below 0.60 were not displayed. Totally, this phylogenetic analysis involved 94 amino acids across 8 animals.

### Test of Variable dN/dS Ratios

To detect selective constraints and positive selection for the three expanded *PyIAP* sub-families (BIRC1, BIRC4, and BIRC5), we used a codon-based models of the CodeML application from the PAML 4.9 package (Yang, 2007) with a maximum likelihood ratio test (LRT). First, The PAML format alignment sequence and Newick format evolution tree file of the three sub-families were loaded, with Num of Threads set to 2, and the consistency of the category name and the alignment sequence name in the evolution tree file were checked through the “Check” button. Then, we used various site models that allow the ω ratio to vary among sites of the expanded *IAP* subfamilies to estimate Non-synonymous (dN) and Synonymous (dS) substitution ratios (dN/dS or ω) and to measure the selective pressure at the protein level, in which a ω of 1, < 1, or > 1 represents neutral evolution, purifying selection/negative selection, or positive selection, respectively. In the site-specific selection analyses, three pairs of codon substitution models (M0 vs. M3, M1a vs. M2a, M7 vs. M8) were conducted (Cheng et al., 2016; Ahmad et al., 2019, 2020; Xun et al., 2020). The model M0 (one ratio) assumes that all sites have the same value, whereas model M3 (discrete) assumes that the ω values of all sites show a simple discrete distribution trend. The model M1a (neutral), allows two classes of codon sites for ω (0 < ω < 1 and ω = 1) and estimates the ratio and ω value of these two types of sites, whereas the model M2a (positive selection), allows one additional site class with ω > 1 and estimates the ratio and ω-value of these three types of sites. The M7 (beta) model assumes that ω (0 < ω < 1) fits a beta distribution, whereas the model M8 (beta and ω) has an extra category with ω > 1. Comparisons between the paired nested site models were performed to evaluate the variation in ω (M0 vs. M3) and to determine the presence of a positively selected class of sites (M1a vs. M2a and M7 vs. M8). After the sites under positive selection were estimated, we applied a Bayes Empirical Bayes (BEB) approach as implemented in PAML 4.9 package to identify the class of sites.

### Expression Profiling of *PyIAP*s After *A. catenella* Exposure

We choose 2-year-old adult Yesso scallops to perform the toxic diet exposure experiment. These scallops were reared and aerated in filtered seawater at 12–13°C for one week to adapt to the laboratory environment. The *A. catenella* (ACDH strain) cells were cultivated (Navarro et al., 2006) and collected at the late exponential growth phase (Garcia-Lagunas et al., 2013) by centrifugation (2500 g/10 min). A total of 3 L of *A. catenella* cells were fed to each scallop once a day, with a final density of 2500 cells/ml. This preliminary experiment of the feeding doses was conducted by setting different cell densities to feed Yesso scallops in order to determine the reasonable food intake. When all 3 L of dinoflagellate cells were eaten up by the exposed scallop in one day, without leftovers in the sea water, the most reasonable cell density was 2500 cells/ml (Hu et al., 2019). In the present study, Yesso scallops were fed continuously, with 3 L dinoflagellate cells each day till their tissues were taken at 0 (control), 1, 3, 5, 10, and 15 days from three scallops for each time point. The hepatopancreases and kidney tissues were collected, rinsed with PBS (1x), immediately frozen with liquid nitrogen, and stored in the refrigerator at −80°C till RNA extraction.

Total RNA was isolated from the sampled hepatopancreas and kidney tissues using phenol- chloroform method. After exposure to toxic ACDH strain, we used RNA-seq data obtained from these tissues to analyze the expression profiles of *PyIAP* genes (Hu et al., 2019). Briefly, we followed the manufacturer’s instructions of NEB Next mRNA Library Prep Kit to construct the RNA-seq libraries, and these libraries were arranged to PE125 sequencing on the Illumina HiSeq 2000 platform. Then, we mapped RNA-seq reads to the *P. yessoensis* genome with the help of Tophat 2.0.9, and the expression of all *PyIAP* genes was normalized and represented in the form of reads per kilobase of exon model per million mapped reads (RPKM). We calculated Fold Change (FC) for each test time point as log2FC among the experimental group (toxin-exposed) with control groups (Supplementary Table 3). The edgeR software (Robinson et al., 2010) was used to analyze the difference of transcriptome data in expression profiles between the experimental group and the control group. For genes screening with significant expression differences, *p* < 0.05 and—log_2_FC— ≥ 1 was used as the criterion (Wang et al., 2019). Finally, MeV 4.90 software^8^ was used to draw a heat map with the log_2_FC values.

## Results and Discussion

### *PyIAP* Genes Identification and Their Genomic Distribution

A total of 47 *IAP* genes were identified from the whole genome of *P. yessoensis* and they were classified into the five sub-families, including BIRC1, BIRC4, BIRC5, BIRC6 and BIRC7 (Table 1). Specifically, more than half of the identified *PyIAP* genes (29 members) belong to the BIRC5 sub-family. Six BIRC1 members and nine BIRC4 members were identified for *PyIAP*s, with the rest three classified into BIRC6 and BIRC7 sub-families, respectively. In comparison with human, from which eight single-copy *IAP* genes known as BIRC1-8 were identified (Salvesen and Duckett, 2002; Vucic, 2008), the *PyIAP*s from BIRC1, BIRC4, and BIRC5 sub-families showed clearly gene expansion, whereas the members from sub-family BIRC2, BIRC3 and BIRC8 are found to be absent in the *P. yessoensis* genome.

**TABLE 1:** Copy numbers of *IAP* sub-families among selected vertebrate and mollusk genomes.

Gene	Human	Mouse	Chicken	Frog	Zebrafish	Octopus	Limpet	Yesso Scallop
BIRC1	1					2		6
BIRC2	1	1			1			
BIRC3	1	1	1					
BIRC4	1	1	1	1			2	9
BIRC5	1	1	1	4	2	1	1	29
BIRC6	1	1			1	1	1	1
BIRC7	1	1	1	2	1	4	5	2
BIRC8	1							
Total	8	6	4	7	5	8	9	47

The basic information of *PyIAP*s including gene ID, GeneBank accession number, gene subfamily, genome position, exon number, CDS length, protein length, BIR domain number, PI value as well as molecular weight were summarized in Supplementary Table 2. The ORF of *PyIAP*s varied from 411 to 14808 bp in length and the protein length ranged from 136 to 4935 amino acids. The exon number of most *PyIAP*s varied from 2 to 25, except for the shortest *PyBIRC5* member (*PY.8149.5*) with a single exon and for the longest *PyBIRC6* member (*PY.8833.5*) that are comprised with 59 exons.

The 47 *PyIAP* genes were found to distribute across 27 scaffolds, and a total of 20 *PyIAP* genes (43%) were found located as gene clusters in form of two or more neighboring members. For example, seven and six *PyIAP*s were found to locate on scaffold9385 and scaffold383, respectively, showing high distribution density. Besides, among eight chromosomes which *PyIAP*s located, chromosome NO.3 and NO.7 were found to be the main chromosomes they distributed, with 28 *PyIAP* genes and seven *PyIAP* genes, respectively. Of note, the *PyIAP*s from high density region were usually from the expanded BIRC4 and BIRC5 subfamily. Taken together, we inferred that the expansion of *PyIAP* genes could be attributed to lineage-specific multiple tandem duplication events. Similar phenomenon has been revealed for *PyHsp70s* (Cheng et al., 2016; Hu et al., 2019), suggesting the involvement of tandem gene duplication in Yesso scallop’s adaptation to the fluctuating and stressful marine environments.

### Conserved Protein Signatures and Phylogenetic Analysis

**FIGURE 1:**
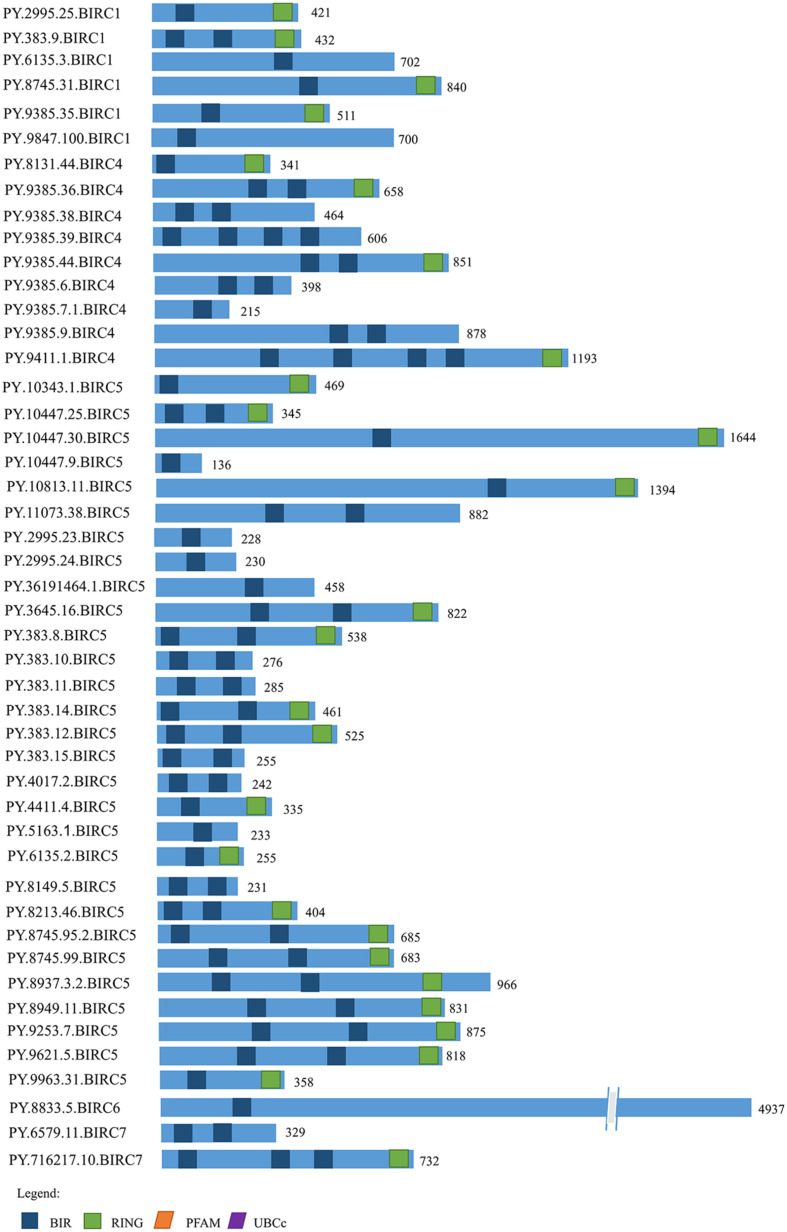
The structure of *IAP* proteins in *Patinopecten yessoensis*. The blue boxes indicate the conserved BIR domains. The green boxes indicate RING domains. The orange box indicates PFAM domain and the purple box indicates UBCc domain. Amino acid length is indicated to the right of each protein (For interpretation of the references to color in this figure legend, the reader is referred to the web version of this article).

**FIGURE 2:**
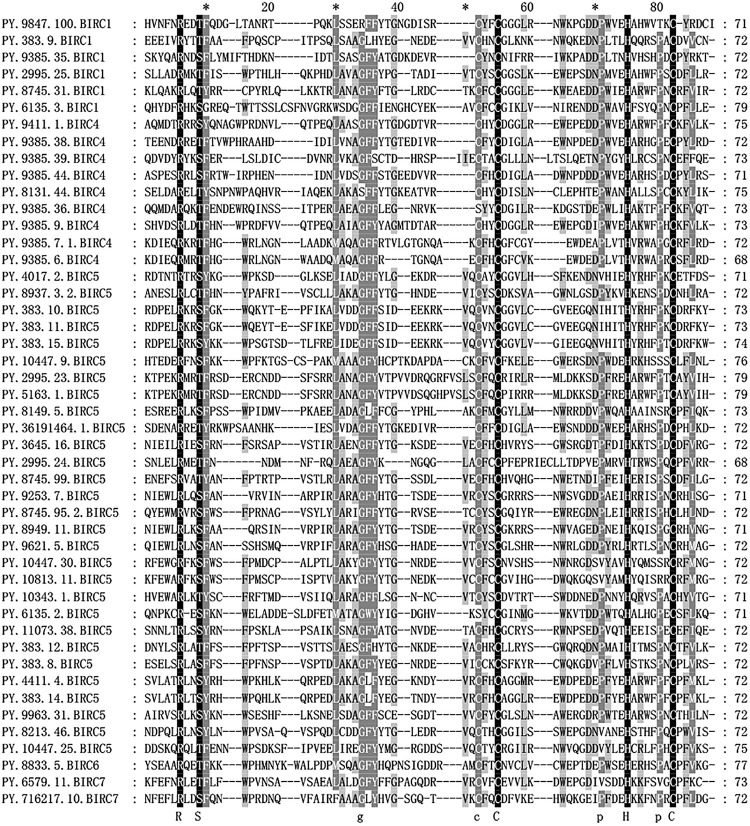
Alignment of *Patinopecten yessoensis* BIR domain sequences. Black indicates that all sequences have the same amino acid at the same site, gray indicates that most sequences have the same amino acid at the same site (For interpretation of the references to color in this figure legend, the reader is referred to the web version of this article).

The IAP proteins are characterized by the presence of Baculovirus IAP Repeat (BIR) at the N-terminal end (Mohamed et al., 2017). According to the analysis of protein structure characteristics (Figure 1), all 47 *PyIAP* proteins possess one or more repeats of BIR domain. According to the multiple sequence alignment, it showed that the *PyBIR* domains were composed of about 68–79 amino acid residues, containing conservative cysteine/histidine sequences (Figure 2). This was consistent with the previous findings that BIRs possessed a great quantity of constant amino acid residues, especially including three conserved cysteines and one histidine that coordinated a zinc ion, which was required to stabilize the BIR fold (Silke and Meier, 2013). Besides BIR domains, 27 *PyIAP*s were found to contain a ring domain at the C-terminus, whereas the longest *PyBIRC6* member (*PY.8833.5*) that mentioned above contain a PFAM as well as a UBCc domain at the C-terminus. Compared with the protein structure of *IAP*s from various species, including viral, yeast, nematode, fly, and several mammalians (Deveraux and Reed, 1999), previous studies found that the number of BIR domains are usually less than three. Three subtypes of BIR domain (BIR1, BIR2, and BIR3) have been identified so far (Mohamed et al., 2017). In *P. yessoensis* we found 2 members of *PyBIRC4* sub-family containing quadruple BIR domains, and previous study had also shown that the BIR domain expanded in the *Crassostrea gigas* (Zhang et al., 2011). It has been reported that although the family defining BIR domain is highly conserved, distinct BIR domains may have different functions (Wang and Lin, 2013). Structure of *IAP*s is closely related to their antiapoptotic ability. The antiapoptotic function of *IAP*s was affected by interplay among the BIR domains with caspases (Wang and Lin, 2013). For instance, the BIR3 domain of *XIAP* in human directly binds to the small subunit of caspase-9, but the BIR2 domain interacts with the active-site substrate binding pocket of caspases-3 and -7 (Takahashi et al., 1998). Thus, the various BIR domain changes in *PyIAP*s, such as the specific quadruple-BIR structure, may contribute to their adaptive functional differentiation in the life activities of scallops which needs to be further studied.

**FIGURE 3:**
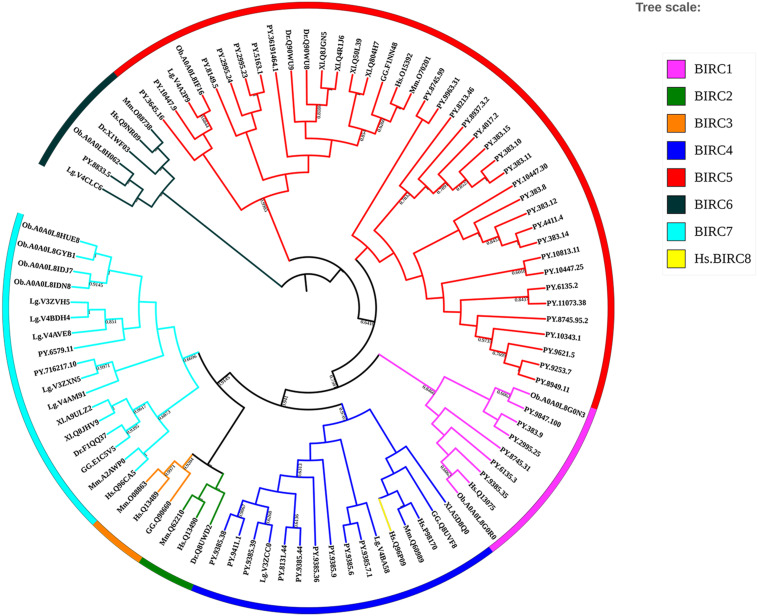
Phylogenetic tree of selected organisms’ *IAP* amino acid sequences. The trees were constructed using NJ method with bootstrapping of 1000 pseudo replicates, the percentage values were converted to 0-1, and scores below 0.60 were not shown (For interpretation of the references to color in this figure legend, the reader is referred to the web version of this article). Species abbreviations: Hs, *Homo sapiens*; Mm, *Mus musculus*; GG, *Gallus gallus*; Xl, *Xenopus laevis*; Dr, *Danio rerio*; Lg, *Lottia gigantean*; Ob, *Octopus bimaculoides*; PY, *Patinopecten yessoensis*.

Inhibitors of apoptosis proteins from eight selected animal species were used to conduct phylogenetic analysis based on NJ method with 1000 bootstrap pseudo-replicates. As shown in Figure 3, all *IAP* proteins could be subdivided into eight proposed *IAP* sub-families, including BIRC 1–8 sub-families. The NJ tree clearly showed three distinct cluster of BIRC1, BIRC4, and BIRC5 sub-families (labeled as purple, navy blue, and red, respectively), which were composed of more than half of *IAP* members from *P. yessoensis*. The orthologous *IAP*s of BIRC6 sub-family were clustered together firstly near the phylogenetic root (black labeled clusters), indicating their ancestor state. Two *P. yessoensis* BIRC7 members together with homologues from other species were first clustered together (light blue), then grouped with the vertebrate BIRC2 (green) and BIRC3 (orange) clusters, implying their closer relationship that may share a same ancestor. Besides, we noticed that, across the species we investigated, there is only one BIRC8 member from human (yellow branch), and it is clustered into BIRC4 sub-family. Previously, it was reported that, within the specific taxonomic lineages, the gene families involved in lineage specific responses to environmental change or microbial attack are usually expanded (Kulmuni et al., 2013; Ellegren, 2014; Zhang et al., 2015). It reminds us that, the obvious gene expansion phenomenon in bivalve *IAP* gene family, especially in the BIRC1, BIRC4, and BIRC5 sub-families, may indicate the existence of an *IAP*s-relevant anti-apoptotic system and making *IAP*s to be good candidates for gene replication and functional diversity research.

### Selective Pressure Analysis of the Expanded *PyIAP*s Sub-Families

Previous study pointed that, through combination of acquisition of new genes and modification of existing genes driven by positive selection, gene replication and expansion could provide support for the emergence of new functional genes (Kulmuni et al., 2013). These new emerged genes could possibly help organisms adapt to complex living conditions (Zhang et al., 2012; Ellegren, 2014). Here, the three expanded sub-families of *PyIAP*s (BIRC1, BIRC4 and BIRC5) in Yesso scallops were analyzed for the selective pressure at protein level, using the Non-synonymous/Synonymous ratio (dN/dS or ω) as an important indicator. As the results of Table 2 showed, variable selection pressure was detected to distribute among codons between *PyBIRC4* and *PyBIRC5* sub-family (*p* < 0.05). In comparison between the M0 and M3 models, the *p*-values of the BIRC1, BIRC4 and BIRC5 sub-families are all 0 (less than 0.05), rejecting the M0 model (single ratio) and supporting the M3 model (discrete), demonstrating that it allows for the three sub-families with different ω ratios. Comparisons of M7 (beta) vs. M8 (beta and ω) revealed statistically significance of permitting positive selection for M8 in *PyBIRC4* and *PyBIRC5* sub-families (*p* < 0.05), suggesting that it provided consistent evidence for the presence of a small proportion of positively selected sites in the *PyBIRC4*s and *PyBIRC5*s. Then, we used the Bayes Empirical Bayes (BEB) approach in PAML to calculate posterior probabilities in order to confirm sites under positive selection (ω > 1) in the M8 model. As shown in Table 2, We have identified six positively selected sites in *PyBIRC4* sub-family (*p* < 0.05) with posterior probability > 95%, suggesting the existence of purifying selection. Scallops are living in highly dynamic marine environment, in order to cope with diverse biotic and abiotic stressors, the scallop’s defense system is expected to change quickly and requires constant adaptive innovations. Gene expansion is a potential source of such innovation (Ellegren, 2014), and the significant expansion of the *IAP* genes in *P. yessoensis* may reflect that the requirement of a certain selection pressure for such genes as a strategy of molecular sequence evolution.

**TABLE 2:** Site model analysis for the expanded *PyIAP* subfamilies.

IAP	No. Copies	LRT statistics (*p* value)(No. Sites Pr > 95%)		
		
		M0 vs. M3 M1a vs. M2a M7 vs. M8		
BIRC1	6	**100.1 (0)**	0 (1)	0.9 (0.624)
BIRC4	9	**137.7 (0)**	0 (1)	**8.5 (0.014) (6)**
BIRC5	29	**253.9 (0)**	0 (1)	**7.9 (0.020)**

*Likelihood ratio test (LRT) is likelihood ratio test statistic for model M0 vs. M3; M1a vs. M2a; M7 vs. M8 from PAML package. Significant results with *P* < 0.05 are highlighted in bold. Pr, posterior probability for sites under positive selection.*

### Expression Profiles of *PyIAP* Genes After *A. catenella* Exposure

**FIGURE 4:**
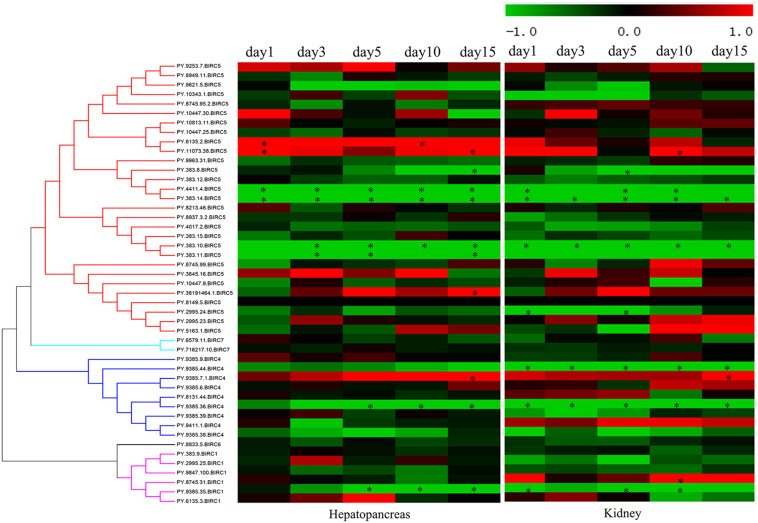
Heatmap of *IAP*s expression in hepatopancreas and kidney of *Patinopecten yessoensis* after ACDH exposure at different time points. * indicated significantly up- and down-regulated *IAP*s from different subfamilies with —log_2_FC— ≥ 1 and *p*-value < 0.05 at each test time point (For interpretation of the references to color in this figure legend, the reader is referred to the web version of this article).

As filter feeding species, scallops can accumulate and transform paralytic shellfish toxins (PSTs) from microalgae. Previous reports have shown that, among all organs, hepatopancreas and kidney are the top-ranked tissues with highest accumulation of PST (Tian et al., 2010; Li et al., 2017). In *P. yessoensis*, compared with hepatopancreas and kidney (∼204 nmol/g), the PSTs accumulation was found far more less in other soft tissues (∼2.8 nmol/g), such as in mantle, gill and muscle, and our preliminary investigation results showed no obvious express change in other soft tissues. Herein, we decided to focus on the responses of *PyIAP*s in hepatopancreas and kidney. It is reported that hepatopancreas in scallop is the organ mainly to accumulate the incoming toxins directly, while in kidney PSTs can change into higher toxic analogs, such as saxitoxin (STX) and neosaxitoxin (NeoSTX), probably as deterrence against predation (Hu et al., 2019). In order to systematically assess the transcriptional responses of *PyIAP*s exposed to toxic dinoflagellates, PST-producing *A. catenella* (strain ACDH) were used as feeding diet and the expression profiling of *PyIAP*s in both hepatopancreas and kidney was analyzed (Figure 4). After exposure, fourteen *PyIAP*s showed significant responses in hepatopancreas and kidney, and all of which were from the expanded subfamilies BIRC1, BIRC4 and BIRC5 (two, three and nine members, respectively). The significant regulation pattern of *PyIAP*s was similar between two tissues, with more than half exhibit expression suppression within three days. Besides, three *PyIAP*s of BIRC5, one member of BIRC1 and one member of BIRC4 showed significant up-regulation with toxic diet. In contrast to hepatopancreas, more acute responses of *PyIAP*s to the toxic stress could be detected in kidney, with seventy percent showed acute down-regulation within one day exposure, implying the possible involvement of these *PyIAP*s in tissue-specific PST tolerance.

In hepatopancreas, the main organ for PST absorption, 46 *PyIAP*s exhibited detectable expression after ACDH exposure, and seven and four *PyIAP*s were significantly down- and up-regulated at least at one test time point, respectively (Figure 4, Table 3 and Supplementary Table 3). Among these 11 *PyIAP*s with significant regulation (| log_2_FC| ≥ 1, *p*-value < 0.05), there are eight members from the most expanded BIRC5 subfamily, with six of them showed acute responses within three days. Of note, two members of them (*PY.4411.4* and *PY.383.14*) were rapidly down regulated on day1 and showed continuous suppression till day15, and specially *PY.383.14* also showed the highest log2FC changes (| log_2_FC| reach to 5.91), suggesting their important roles facing toxin accumulation. Besides, three *PyBIRC5*s were significantly induced, with two members (*PY.6135.2*, *PY.11073.38*) being up-regulated on day1 and one member (*PY.36191464.1*) chronically up-regulated on day15. Other than the BIRC5 subfamily, there were one member of BIRC1 and two members of BIRC4 presented chronic responses, being down-regulated after 5 days exposure or up-regulated on day15.

**TABLE 3:** Summary of *PyIAPs* expression in hepatopancreas and kidney after exposure to toxic dinoflagellate ACDH.

ACDH	Hepatopancreas						Kidney					
	**1day**	**3day**	**5day**	**10day**	**15day**	**sum**	**1day**	**3day**	**5day**	**10day**	**15day**	**sum**
BIRC5(29)	2-2	0-4	0-4	1-3	2-5	**3-5**	0-4	0-2	0-5	1-3	0-2	1-5
BIRC7(2)	−	−	−	−	−	**−**	−	−	−	−	−	−
BIRC4(9)	−	−	0-1	0-1	1-1	**1-1**	0-2	0-2	0-2	0-2	1-2	1-2
BIRC6(1)	−	−	−	−	−	**−**	−	−	−	−	−	−
BIRC1(6)	−	−	0-1	0-1	0-1	**0-1**	0-1	−	0-1	1-1	−	1-1
sum	2-2	0-4	0-6	1-5	3-7	4-7	0-7	0-4	0-8	2-6	1-4	3-8

*The numbers indicated significantly up- and down-regulated *IAPs* from different subfamilies with —log_2_FC— ≥ 1 and *p-*value < 0.05 at each test time point.*

In kidney, where PSTs could be transformed to derivatives with higher toxicity, also there were 46 *PyIAP*s expressed after ACDH exposure, and eight and three *PyIAP*s were significantly down-regulated and up-regulated at least at one test time point, respectively (Figure 4, Table 3 and Supplementary Table 3). Notably, in comparison with hepatopancreas, more acute down regulations of *PyIAP*s in kidney could be detected. Seven *PyIAP*s, including four *PyBIRC5*s, two *PyBIRC4*s and one *PyBIRC1*, reduced their expression significantly within one day exposure and four of them showed continuous suppression till day15, reflecting the sensitive and intensive responses to toxic effects. Especially, *PY.383.14* again showed quite high log2FC changes (| log_2_FC| reach to 5.19), just like it’s performance in hepatopancreas, reinforcing evidence linking its participation during toxic adaption. Besides, three members including *PyBIRC1* (*PY.8745.31*), *PyBIRC4* (*PY.9385.7.1*) and *PyBIRC5* (*PY.11073.38*) were found significantly induced in kidney after five days exposure.

After ACDH exposure, in the two major toxic organs of *P. yessoensis*, most of the *IAP* genes with significant altered expression levels were from the expanded BIRC5 and BIRC4 subfamily. Out of the nine significantly down-regulated *PyIAP*s with toxic diet, four genes are located on scaffold383 and three genes located on scaffold9385. Interestingly, on both of these two scaffolds, a pair of *PyIAP*s (*PY.383.10*/*383.11* and *PY.9385.35*/*9385.36*) that possess the adjacent position could be found, suggesting the *IAP* genes duplication and expansion in *P. yessoensis* could have important effects on scallop’s toxin resistance.

## Conclusion

In this study, a comprehensive identification and characterization of *IAP* genes were performed for the first time in Yesso scallop *P. yessoensis*. Forty-seven *PyIAP*s from five subfamilies were identified, and significant expansion of subfamilies BIRC4, BIRC5 that under the pressure of purifying selection were revealed. After exposure to PST-producing dinoflagellates, *A. catenella*, fourteen *PyIAP*s showed significant responses in hepatopancreas and kidney, and two, three and nine members were from the expanded subfamilies BIRC1, BIRC4 and BIRC5, respectively. Significant regulation pattern of *PyIAP*s was similar between two tissues, with more than half exhibit expression suppression within three days, and most of which are usually located as gene clusters that distributed on the same scaffold, suggesting the *PyIAP*s duplication and expansion could have important effects on scallop’s toxin resistant. Besides, in comparison with hepatopancreas, more acute responses of *PyIAP*s to the toxic stress could be detected in kidney, implying the possible involvement of these *PyIAP*s in tissue-specific PST tolerance. These findings could assist in a better understanding of the adaptive expansion of bivalve *IAP* genes to the marine environments with algae derived biotoxins, and providing good candidate members of *IAP* genes for their further functional study in marine bivalves.

## Data Availability Statement

The original contributions presented in the study are included in the article/Supplementary Material, further inquiries can be directed to the corresponding author/s.

## Author Contributions

SL, SW, and ZB conceived and designed the study. XZ, FZ, YW, and NH performed the experiments. XZ, XC, and XD participated in data analysis. XZ, SL, and XH wrote the manuscript. All authors contributed to the article and approved the submitted version.

## Conflict of Interest

The authors declare that the research was conducted in the absence of any commercial or financial relationships that could be construed as a potential conflict of interest.

## Footnotes

1 https://www.ncbi.nlm.nih.gov/

2 https://www.uniprot.org/

3 http://www.ncbi.nlm.nih.gov/orffinder/

4 http://smart.embl-heidelberg.de/

5 https://www.expasy.org/

6 http://gsds.gao-lab.org/index.php

7 http://itol.embl.de/

8 http://mev.tm4.org

## Supplementary Material

Supplementary Table 1 *IAP* protein sequences accession ID of different species used in this study. Sequences were retrieved from Uniprot and NCBI database.

Supplementary Table 2 Summarized information of *IAP* genes identified in *P. yessoensis* genome.

Supplementary Table 3 Different expression of *PyIAP*s in hepatopancreas and kidney after *A. catenella* exposure analyzed using edgeR package.
